# Sex-Specific Differences in Gut Microbial Composition and Metabolism of Jiangshan Black Pigs

**DOI:** 10.3390/microorganisms13112551

**Published:** 2025-11-07

**Authors:** Yanan Zhang, Xian Wu, Dan Song, Panlin Wang, Haifeng Wang, Xiangchen Li

**Affiliations:** 1 Key Laboratory of Applied Technology on Green-Eco-Healthy Animal Husbandry of Zhejiang Province, College of Animal Science and Technology & College of Veterinary Medicine, Zhejiang A&F University, Hangzhou 311300, China; yananzhang@zafu.edu.cn (Y.Z.); wuxian723@163.com (X.W.); songdan2020@zafu.edu.cn (D.S.); wangpl2021@zafu.edu.cn (P.W.); 2 The Key Laboratory of Molecular Animal Nutrition, Ministry of Education, College of Animal Science, Zhejiang University, Hangzhou 310058, China

**Keywords:** sex discrepancy, gut microbiota, microbial metabolism, microbial function, Jiangshan black pig

## Abstract

The gut microbiota plays a vital role in regulating the host’s physiological functions, including metabolism and immunity. The microbial composition and metabolism are modulated by multiple factors; host sex is an important yet under-explored determinant. To investigate the sex-dependent differences in the gut microbiota within the small and large intestine, sixteen somatic mature Jiangshan black pigs (eight males and eight females) were analyzed. The ileal and colonic microbial community and metabolites were profiled using 16S rRNA gene high-throughput sequencing and gas chromatography. Distinct sex-related discrepancies were observed in both the microbial composition and metabolism of the ileum and colon. In the ileum, compared with the male group, the female group exhibited higher abundances of Unclassified *Chloroplast* and *Pseudomonas* but a lower abundance of *Romboutsia* (adjusted *p* < 0.05). Functional prediction indicated enrichment in amino acid metabolism pathway in females, with more copy numbers of genes encoding key enzymes for propionate (*mmdA*) generation and elevated valerate levels (*p* < 0.05). In the colon, compared with the male group, the female group showed higher abundances of *Streptococcus*, *Phascolarctobacterium,* and *Prevotella* spp. and lower abundances of *Eubacterium coprostano-ligenes group*, *Blautia*, *Christensenellaceae R-7 group*, and *Ruminococcus* (adjusted *p* < 0.05). Additionally, the female group had more copies of genes *mmdA* and *LcdA* (associated with lactate production), along with higher concentrations of propionate and lactate (*p* < 0.05). Correlation analysis between microbial metabolites and sex-biased bacteria further revealed that the SCFA concentration positively correlated with *Prevotella* spp. and negatively correlated with *Romboutsia*, *Christensenellaceae R-7 group*, and *Blautia*. Collectively, these findings highlight the pronounced sex-dependent discrepancies in the microbial composition and metabolism within the small and large intestines of Jiangshan black pigs, providing new insights for precisely modulating the microbiota community and metabolism in pigs according to sex.

## 1. Introduction

The mammalian gastrointestinal tract harbors a large, complex, and dynamically changing community of microorganisms exceeding 100 trillion microbial cells [1]. Microbial communities and metabolites could directly or indirectly contribute to various physiological functions of the host, such as growth and development, nutrient metabolism, immunity, and signal transmission [2,3], thereby maintaining host health and growth.

The gut microbial community serves as an intricate and heterogeneous ecosystem that is easily influenced by dietary composition, drugs, environment, and host inheritance [4,5,6]. In recent years, a growing number of studies have found that the host sex plays an important role in shaping the gut’s microbial community [7,8]. One human study revealed that there were significant differences in the gut microbial composition between women and men, with women exhibiting higher microbial diversity than men [9]. For instance, the genera *Prevotella*, *Fusobacterium*, *Megasphaera*, and *Megamonas* were enriched in the gut of men; *Akkermansia*, *Ruminococcus*, and *Bifidobacterium* were enriched in the gut of women [10]. Similar results have also been obtained in mice [11]. Furthermore, compared with normal male mice, sexually mature castrated male mice exhibited a gut microbial composition similar to that of females [12]. In pigs, some studies have revealed that the gut microbial composition exhibited sex-specific characteristics [13,14]. For example, the relative abundances of *Escherichia*, *Roseburia*, and *Veillonella* were higher in the feces of male Duroc pigs, while *Bacteroides* and *Treponema* were higher in female Duroc pigs [13]. Moreover, the host sex has significant influences on microbial metabolism [12]. A previous study reported that the metabolic activity of *Lactobacillus* exhibited a clear sex bias, with higher levels of propionate, butyrate and lactate in females, thus modulating gut health and energy metabolism [15]. The gut microbiota of female and male mice showed significant differences in lipid-metabolism-related genes. Compared with the male mice, the concentrations of total and primary bile acids were higher in the gut of female mice [16], which were closely related to higher relative abundances of *Lachnoclostridium* and *Escherichia Shigella* in the duodenum [17]. Sex-related differences are also evident in nitrogenous compound metabolism: Gao et al. reported higher colonic leucine and lower phenylalanine and tyrosine levels in female versus male mice [18]. Despite these findings, the understanding of how host sex influences the microbial composition and metabolism in the gut of pigs remains limited.

Extensive researches have demonstrated that the composition and metabolism of the microbiota in the gastrointestinal tract of pigs varies between the small intestine and large intestine [4,19]. In growing pigs, Firmicutes and Proteobacteria were dominant in the ileum, while Firmicutes and Bacteroidota were dominant in the colon [20]. Previous studies have found that the microbes from the small intestine and large intestine had different roles in amino acid metabolism and carbohydrate metabolism [21,22]. These findings revealed that the microbiota in different intestinal compartments perform unique functional roles. It is widely recognized that the microbiota in the small intestine (10^4^–10^7^ CFU/mL) is substantially smaller than that in the large intestine (10^11^–10^12^ CFU/mL) [23], and therefore the microbial community of the small intestine has received relatively limited attention. However, the impact of host sex on the microbiota in different intestinal compartments remains unclear.

Jiangshan black pig, a local Chinese breed pig (from Jiangshan city, Zhejiang Province, China), is known for its rich genetic background and excellent germplasm characteristics, such as roughage resistance, strong maternity, high livability and adaptability, and high disease resistance. Despite these valuable characteristics, few studies have investigated the gut microbiota of the Jiangshan black pig, especially in relation to sex-specific discrepancies. Thus, this study aimed to characterize the differentiation in the composition and metabolism of microbes from both the ileum and colon between the sexes of Jiangshan black pigs using 16S rRNA gene high-throughput sequencing and gas chromatography. The results showed that sex-associated disparities exist in the microbial composition and metabolism in the ileum and colon. These findings provide novel insights into how host sex influences the gut microbial community in different intestinal regions of the Jiangshan black pig and show that sex may be a significant factor in determining the microbial community.

## 2. Materials and Methods

### 2.1. Animals and Sampling

**Figure 1:**
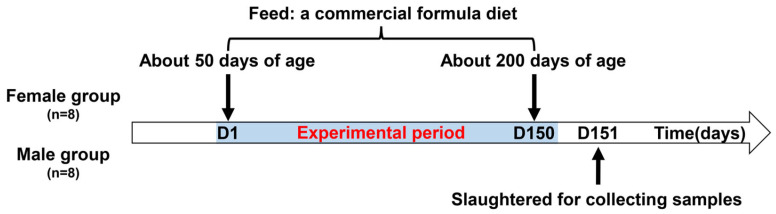
A flow chart of the experimental design in this study.

A total 16 healthy weaned Jiangshan black pigs aged approximately 50 days were obtained from a commercial farm in Jiangshan City (Zhejiang Province, China). According to sex, the pigs were divided into 2 groups, a female group and a male group (intact boars) (*n* = 8). All the pigs were housed individually in a metal pen with ad libitum access to feed (a commercial formula diet) and clean water; the diet’s details are listed in Table 1. The pigs were raised until about 200 days of age to ensure somatic maturity (body weight exceeds 50 kg). The final body weight and age of the female pigs were 52.0 ± 4.72 kg and 192 ± 8 days, respectively; those of the male pigs were 59.6 ± 6.09 kg and 205 ± 3 days, respectively. Then, all pigs were slaughtered for sample collection. After slaughtering, the gastrointestinal tract of the pig was removed immediately, and intestinal segments were identified and ligated. The ileum was from the jejunal–ileal junction to the ileal–cecal junction; the colon was from the cecal–colonic junction to the rectal–anal junction. Subsequently, the intestinal segments were cut open longitudinally to collect the digesta using a sterile 5 mL tube and immediately stored at −20 °C for the analysis of the microbial composition and metabolites. A flow chart of the experimentation is shown in Figure 1.

### 2.2. DNA Extraction, Illumina MiSeq Sequencing, and Data Processing

Total microbial genomic DNA was extracted from 0.5 g of ileal or colonic digesta samples according to a previous study by Dai et al. [24]. Briefly, the samples were mixed with sterile cetyltrimethyl ammonium bromide (CTAB) buffer using bead-beating for splitting the cell wall of the bacteria. The DNA was extracted by the phenol–chloroform method and finally suspended in TE buffer. The quantity of DNA was measured using a NanoDrop spectrophotometer (Thermo Scientific, Wilmington, DE, USA) and stored at −80 °C until further processing.

The V3 to V4 regions of the bacterial 16S rRNA gene were amplified using a universal forward primer, 341F (5′-CCTAYGGGRBGCASCAG-3′), and a reverse primer, 806R (5′-GGACGGACTACNNGGGTATCTAAT-3′). The amplicons were purified using a DNA Gel Extraction Kit according to the manufacturer’s instructions (Axygen Biosciences, Union City, CA, USA). Purified amplicons were pooled in equimolar and paired-end-sequenced on an Illumina MiSeq PE250 platform according to the standard protocols [25].

The raw sequence data from 16S rRNA gene MiSeq sequencing were analyzed using QIIME 2 (version 2020.2). Briefly, raw reads were qualified, denoised, classified, and counted using DADA2 to generate an amplicon sequence variant (ASV) table [26]. The ASVs with a minimum of four reads and present in more than one sample were retained. The Silva 138 database was used to annotate the ASVs [27]. The α-diversity (Chao1 richness estimators, Shannon diversity indices) and principal coordinate analysis (PCoA) based on the Bray–Curtis distance metrics were assessed using MOTHUR software (version 1.48.0) [28]. Significant and differential ASVs between 2 groups were determined by linear discriminant analysis (LDA) effect size (LDA score > 2 as discriminant taxa) [29].

### 2.3. Functional Prediction of Microbial Metagenomes

To predict the functional capacity of the gut microbiome, metagenomics function based on 16S rRNA gene was predicted with PICRUSt [30]. The gene categories were predicted at levels 2 and 3 of the Kyoto Encyclopedia of Genes and Genomes (KEGG) ontology groups [31]. Then, the predicted KEGG ontologies were classified into each KEGG pathway, the relative abundance of which was calculated. Significant differences in key KEGG pathways were analyzed between the groups.

### 2.4. Quantifying Copy Numbers of Functional Bacterial Groups and Genes by qPCR

To further reflect the effects of sex on microbial community and function in the ileum and colon, the copy numbers of functional bacterial groups and genes encoding key enzymes involved in propionate and butyrate generation and lactate utilization were quantified by a real-time PCR assay on a QuantStudio5 Flex detection system (Thermo Fisher Inc., Waltham, MA, USA) according to the manufacturer’s instructions using a SYBR Green Pro Taq HS Premix kit (Cat. AG11718, Accurate Biology Co., Ltd., Changsha, China). The detailed procedures, including reaction mixtures, PCR conditions, clone library constructions, and standard curve preparation, were performed according to previous methods [32]. The bacterial groups included total bacteria, Firmicutes, Bacteroidota, *Lactobacillus*, *Bifidobacterium*, *Streptococcus*, *Coprococcus eutactus*, *Faecalibacterium prausnitzii*, *Megasphaera elsdenii*, and *Escherichia coli*. And, the functional genes, including methylmalonyl-CoA decarboxylase (*mmdA*) and butyryl-CoA:acetate CoA-transferase (*BCoAT*), were used for quantifying propionate- and butyrate-producing enzymes, respectively; lactoyl-CoA dehydratase (*LcdA*) was used for quantifying lactate-utilizing enzymes. All the primers used in this study are listed in Table 2.

### 2.5. Determination of Microbial Metabolites

Short-chain fatty acids (SCFAs) in the ileal and colonic digesta samples were detected by gas chromatography according to a previous method [42]. Briefly, the digesta samples were weighted into a sterile centrifuge tube, double distilled water was added, and the samples were vortexed and centrifuged. Then, the supernatant was mixed with 25% (*w*/*v*) metaphosphoric acid. After allowing to stand overnight at −20 °C, the supernatant was centrifuged and filtered with a 0.22 μm filter for measurement. Lactate was measured using a commercial kit according to the manufacturer’s instructions (Nanjing Jiancheng Biological Engineering Institute, Nanjing, China). The concentration of NH_3_-N and microbial protein (MCP) in the digesta was assayed by spectrophotometer colorimetry according to the methods in previous studies [43,44].

### 2.6. Statistical Analysis

All data were analyzed by using SPSS 20.0 (SPSS Inc., Chicago, IL, USA). The statistical differences in bacterial abundance and the KEGG pathways were analyzed using the Mann–Whitney *U* test. To avoid type I errors during microbiota analysis, the *p*-value was adjusted with the Benjamini–Hochberg false discovery rate (FDR) multiple-testing correction; *q* (adjusted *p*-value) < 0.05 was regarded as statistically significant. The data of copy numbers of functional bacterial groups; genes; microbial metabolites, including SCFAs, lactate, NH_3_-N, and MCP, were analyzed with Student’s *t*-test to detect significant differences between the two groups. A *p*-value < 0.05 was considered statistically significant. All data were visualized using GraphPad Prism version 10.0 (GRAPHPAD Software, San Diego, CA, USA).

## 3. Results

Throughout the whole experiment, none of the pigs had diarrhea or other health problems (no pigs received medication).

### 3.1. The Effect of Sex on the Microbial Community in the Ileum and Colon

**Figure 2:**
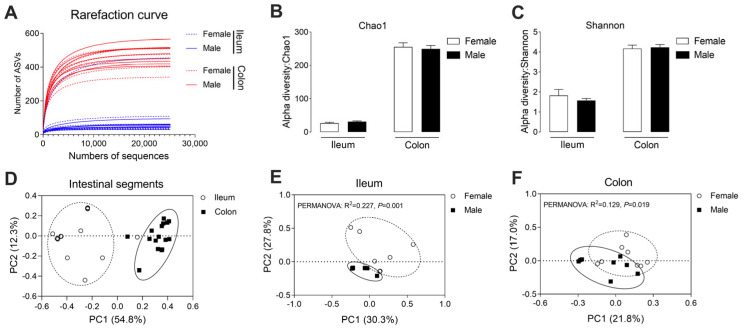
The microbial community structure in the ileum and colon. (**A**) The rarefaction curve; (**B**,**C**) the alpha diversity of the microbial community in the gut: Chao 1 index, Shannon index. Data are presented as mean ± SEM, *n* = 8; (**D**) PCoA of the microbiota in different intestinal segments; (**E**,**F**) PCoA of the microbiota in the ileum and colon, respectively.

The effects of sex on the ileal and colonic microbial composition were revealed by 16S rRNA high-throughput sequencing. In total, 805,056 reads were obtained after the quality control, with an average of 25,158 reads per sample. The rarefaction curve of the individual samples based on the ASV numbers showed we had sufficient sequences for further analysis (Figure 2A). The alpha diversity of the microbial community, including Chao1 and Shannon indices, did not differ significantly between the female group and male group in either the ileum or colon (Figure 2B,C). For the beta diversity of the microbial community, principal coordinate analysis (PCoA) showed a clear distinct between the ileum and colon (Figure 2D) and between the female group and male group in the ileum and colon (Figure 2E,F), suggesting that the sex had a significant influence on the microbial structure in the various intestinal segments.

**Figure 3:**
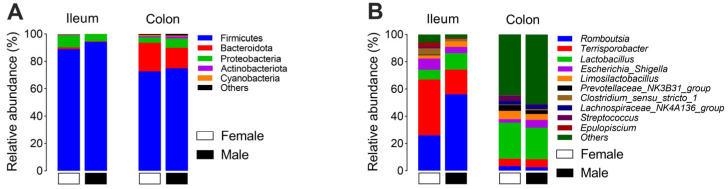
The relative abundance of microbial composition at the phylum (**A**) and genus (**B**) levels in both the ileum and colon.

**Figure 4:**
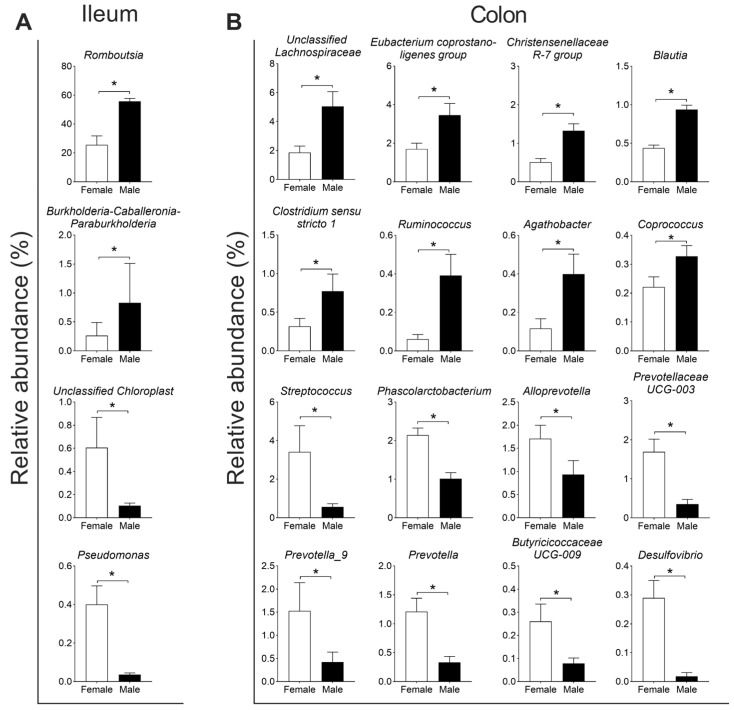
The significantly different genera in the ileum (**A**) and colon (**B**). Data are presented as mean ± SEM, *n* = 8, * *q* < 0.05.

For the composition of the microbiota, Firmicutes (91.6%) and Proteobacteria (7.3%) were the two dominant phyla in the ileum, and Firmicutes (73.7%) and Bacteroidota (18.1%) were the two dominant phyla in the colon (Figure 3A). At the genus level, *Romboutsia*, *Terrisporobacter*, and *Lactobacillus* were the three dominant genera in the ileum (Figure 3B). Further differential analysis revealed that the relative abundances of *Romboutsia* and *Burkholderia–Caballeronia–Paraburkholderia* (BCP) were higher, while Unclassified *Chloroplast* and *Pseudomonas* were lower in the male group than in the female group (*q* < 0.05, Figure 4A). In the colon, *Terrisporobacter*, *Lactobacillus*, and *Escherchia_Shigella* were the three dominant genera (Figure 3B); and compared with the female group, the male group showed higher abundances in the genera *unclassified Lachnospiraceae*, *Eubacterium coprostano-ligenes group*, *Christensenellaceae R-7 group*, *Blautia*, *Clostridium sensu stricto 1*, *Ruminococcus*, *Agathobacter*, and *Coprococcus*, with lower abundances in genera *Streptococcus*, *Phascolarctobacterium*, *Alloprevotella*, *Prevotellaceae UCG-003*, *Prevotella_9*, *Prevotella*, *Butyricicoccaceae UCG-009*, and *Desulfovibrio* (*q* < 0.05, Figure 4B).

**Figure 5:**
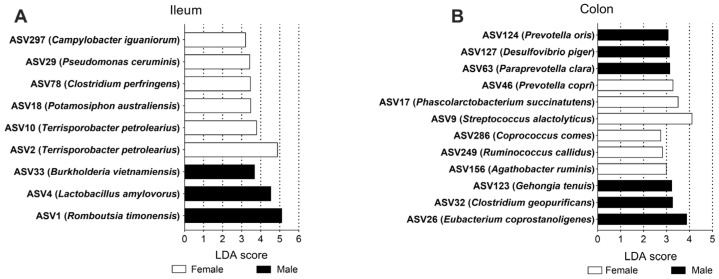
The significantly different ASVs based on LDA in the ileum (**A**) and colon (**B**). ASV: amplicon sequence variants; LDA: linear discriminant analysis.

At the ASV level, in the ileum, nine ASVs showed differential abundances between the female group and male group, including *Campylobacter iguaniorum*, *Pseudomonas ceruminis*, *Clostridium perfringens*, *Potamosiphon australiensis*, and *Terrisporobacter petrolearius*, which were higher in the female group; *Burkholderia vietnamiensis*, *Lactobacillus amylovorus*, and *Romboutsia timonesis* were higher in the male group (Figure 5A). In the colon, a total of twelve ASVs were different in abundance between the groups, with *Prevotella copri*, *Phascolarctobacterium succinatutens*, *Streptococcus alactolyticus*, *Coprococcus comes*, *Ruminococcus callidus* and *Agathobacter ruminis* being higher in the female group; *Prevotella oris*, *Desulfovibrio piger*, *Paraprevotella clara*, *Gehongia tenuis*, *Clostridium geopurificans*, and *Eubacterium coprostanoligenes* were higher in the male group (Figure 5B). These results indicated that sex could serve as one important factor influencing the composition of the gut microbiota.

### 3.2. The Effect of Sex on the Microbial Function in the Ileum and Colon Based on Metagenomics Prediction

**Figure 6:**
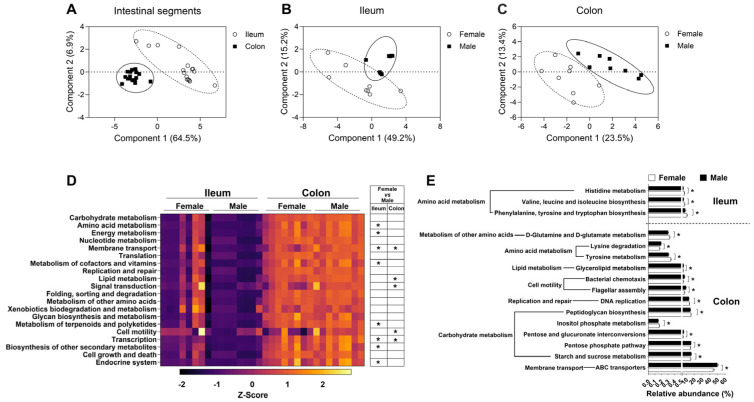
The microbial functions based on the metagenomic prediction results. (**A**) PLS-DA of the microbial function in intestinal segments; (**B**,**C**) PLS-DA of the microbial function in ileum and colon, respectively; (**D**) the heatmap of microbial functional metabolism-related pathways based on KEGG database at level 2 KOs; (**E**) significantly different bacterial metabolic pathways at level 3 KOs. Data are presented as mean ± SEM, *n* = 8, * *q* < 0.05.

To reveal the differentiation in the microbial function between the female group and male group in the ileum and colon, microbial function was predicted with the KEGG database. Partial least-squares discriminant analysis (PLS-DA) demonstrated significant differences in microbial function between the ileum and colon (Figure 6A). Moreover, the microbial function between the female group and male group was also significantly different in both the ileum and colon (Figure 6B,C).

At KEGG level 2 of KOs, the top 20 functional pathways with relative abundance differences between the ileum and colon were visualized with a heatmap (Figure 6D). In the ileum, the relative abundances of KOs predicted to be involved in amino acid metabolism, energy metabolism, membrane transport, metabolism of cofactors and vitamins, metabolism of terpenoids and polyketides, and transcription and endocrine system were higher in the female group than in the male group (*q* < 0.05, Figure 6D). In the colon, the relative abundances of KOs predicted to be involved in membrane transport, lipid metabolism, signal transduction, cell motility and transcription were higher in the male group than in the female group (*q* < 0.05, Figure 6D).

At KEGG level 3 of KOs, the microbial functional pathways were further analyzed, as shown in Figure 6E. In the ileum, the relative abundances of KOs predicted to be involved in histidine metabolism; valine, leucine, and isoleucine biosynthesis; phenylalanine, tyrosine, and tryptophan biosynthesis were higher in the female group than in the male group (*q* < 0.05, Figure 6E). In the colon, the relative abundances of KOs predicted to be involved in D-glutamine and D-glutamate metabolism, tyrosine metabolism, peptidoglycan biosynthesis, and inositol phosphate metabolism were higher in the female group; lysine degradation, glycerolipid metabolism, bacterial chemotaxis, flagellar assembly, pentose and glucuronate interconversions, pentose phosphate pathway, starch and sucrose metabolism and ABC transporters was higher in the male group (*q* < 0.05, Figure 6E). These findings suggested that sex also exerted an influence on the function of the gut microbiota.

### 3.3. The Effect of Sex on the Functional Bacterial Groups and Functional Genes in the Ileum and Colon

**Figure 7:**
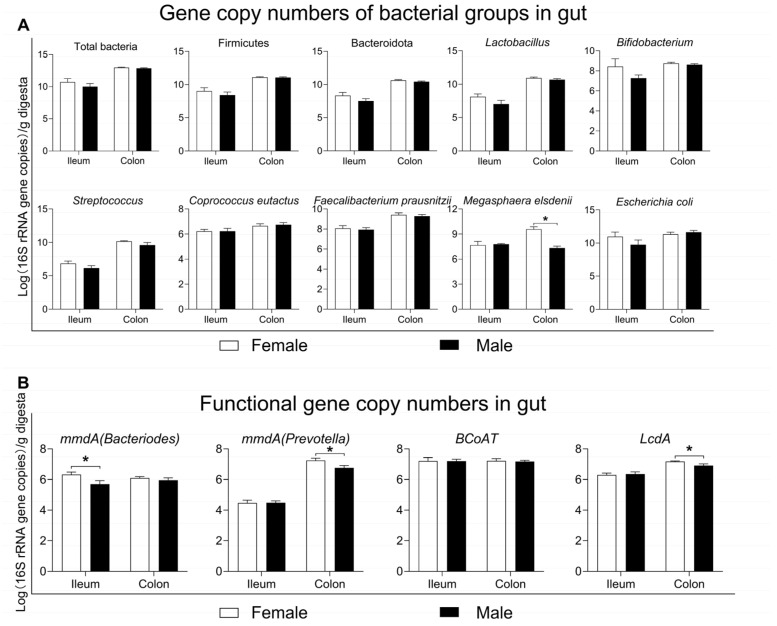
Quantification of function bacterial groups and genes in the gut.: (**A**) 16S rRNA gene copy numbers of bacterial groups in the ileum and colon; (**B**) copy numbers of functional genes encoding key enzymes involved in SCFA generation (*mmdA* and *BCoAT*) and lactate utilization (*LcdA*). Data are presented as mean ± SEM, *n* = 8, * *p* < 0.05. *mmdA*: methylmalonyl-CoA decarboxylase; *BCoAT*: butyryl-CoA:acetate CoA-transferase; *LcdA*: lactoyl-CoA dehydratase.

Regarding 16S rRNA high-throughput sequencing, only the relative abundance of microbes was considered when analyzing the microbial composition. Therefore, qPCR was further performed to quantify the changes in the major functional bacterial groups between the female and male pigs. Firstly, the numbers of total bacteria did not differ significantly between the two groups in either the ileum or colon (*p* > 0.05, Figure 7A). At the taxonomy level, both in the ileum and colon, there were no differences in the numbers of the dominant phyla (Firmicutes and Bacteroidota), common genera (*Lactobacillus*, *Bifidobacterium* and *Streptococcus*), and butyrate-producing species (*Coprococcus eutactus*, *Faecalibacterium prausnitzii*) or harmful species (*Escherichia coli*) between the two groups (*p* > 0.05, Figure 7A). However, the numbers of *Megasphaera elsdenii* were significantly lower in the male group than in the female group in the colon (*p* < 0.05, Figure 7A).

In addition to measuring the numbers of bacterial groups in the gut, the copy numbers of functional genes encoding key enzymes involved in SCFA biosynthesis were also assayed, as shown in Figure 7B. In the ileum, the copy numbers of the functional gene *mmdA* (*Bacteriodes*), involved in propionate production, were lower in the male group than in the female group (*p* < 0.05, Figure 7B). In the colon, the copy numbers of functional genes *mmdA* (*Prevotella*) and *LcdA*, involved in propionate production and lactate utilization, respectively, were also lower in the male group than in the female group (*p* < 0.05, Figure 7B), hinting that the female group possessed a greater metabolic potential for propionate production by the gut microbiota.

### 3.4. The Effect of Sex on the Microbial Metabolites in the Ileal and Colonic Digesta

**Figure 8:**
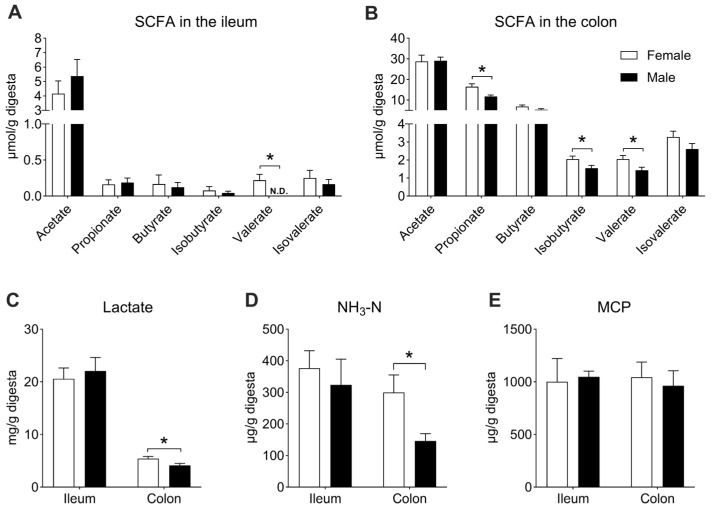
The concentrations of microbial metabolites in the gut. (**A**) SCFA concentration in the ileum; (**B**) SCFA concentration in the colon; (**C**) lactate concentration in the ileum and colon; (**D**) NH_3_-N concentration in the ileum and colon; (**E**) MCP concentration in the ileum and colon. Data are presented as mean ± SEM, *n* = 8, * *p* < 0.05. “N.D.” indicates not detected.

Undigested nutrients in the diet could serve as nutritional substrates to be fermented by gut microbes, producing a variety of metabolites, such as SCFAs, lactate, NH_3_-N, and MCP. In the ileum, the concentration of valerate was higher in the female group than in the male group (*p* < 0.05, Figure 8A), whereas no significant differences were observed in other SCFAs, lactate, NH_3_-N, or MCP between the two groups (*p* > 0.05, Figure 8A–E). In the colon, compared with the female group, the concentrations of propionate, isobutyrate, valerate, lactate, and NH_3_-N were lower in the male group (*p* < 0.05, Figure 8B–D). There was no difference between the two groups in the MCP concentration (*p* > 0.05, Figure 8E).

**Figure 9:**
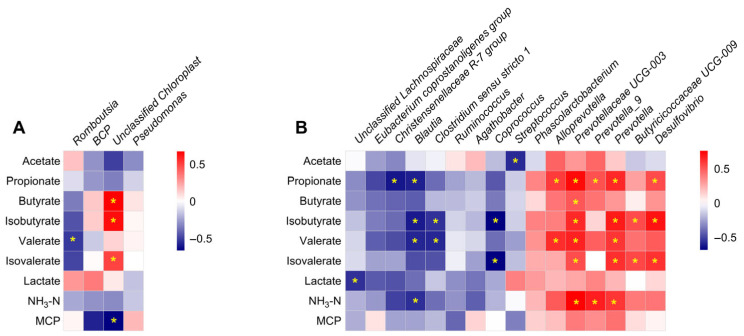
Correlation analysis of significantly different relative abundances of bacteria at the genus level and microbial metabolites concentrations in the ileum (**A**) and colon (**B**). * *p* < 0.05.

To further elucidate the relationship between the gut microbial community and microbial metabolites, Spearman’s correlation analysis was performed based on the relative abundance of bacterial groups and microbial metabolite concentrations. In the ileum, the relative abundances of *Romboutsia* and Unclassified *Chloroplast* negatively correlated with valerate and MCP concentrations, respectively (Figure 9A). In contrast, the relative abundance of Unclassified *Chloroplast* positively correlated with butyrate, isobutyrate, and isovalerate (Figure 9A). In the colon, the relative abundance of *Streptococcus* negatively correlated with acetate concentration, while the relative abundances of *Christensenellaceae R-7 group* and *Blautia* negatively correlated with propionate concentration. In contrast, *Alloprevotella*, *Prevotellaceae UCG-003*, *Prevotella_9*, *Prevotella*, and *Desulfovibrio* were positively correlated with propionate concentration (Figure 9B). The relative abundance of *Prevotellaceae UCG-003* positively correlated with butyrate content, whereas the relative abundances of *Blautia*, *Clostridium sensu stricto 1*, and *Coprococcus* negatively correlated with isobutyrate content. Meanwhile, *Prevotellaceae UCG-003*, *Prevotella*, *Butyricicoccaceae UCG-009*, and *Desulfovibrio* were positively correlated with isobutyrate content. Additionally, the relative abundances of *Blautia* and *Clostridium sensu stricto 1* negatively correlated with valerate content, while *Alloprevotella*, *Prevotellaceae UCG-003*, and *Prevotella* were positively correlated with valerate content (Figure 9B). The relative abundance of *Coprococcus* negatively correlated with isovalerate concentration, while *Prevotellaceae UCG-003*, *Prevotella*, *Butyricicoccaceae UCG-009*, and *Desulfovibrio* were positively correlated with it (Figure 9B). Finally, the relative abundance of Unclassified *Lachnospiraceae* was negatively correlated with lactate concentration; the relative abundance of *Blautia* negatively correlated with NH_3_-N concentration, while *Prevotellaceae UCG-003*, *Prevotella_9*, and *Prevotella* were positively correlated with NH_3_-N concentration (Figure 9B).

## 4. Discussion

The gut microbiota has been reported to contribute significantly to host metabolism and immune functions, thereby maintaining intestinal homeostasis and host health. The differences in the intestinal microbial composition and metabolism mainly depend on host genotype, diet, and environment [6,45,46]. In addition to these factors, sex has recently been recognized as an important contributor to microbiota variability [7]. In this study, we characterized the sex-specific discrepancies in microbial composition and metabolism in different intestinal compartments of Jiangshan black pigs. The results showed that the sex-related differences in the microbial composition and metabolism were significant in the ileum and colon of Jiangshan black pigs. Some sex-biased bacteria were identified. Meanwhile, the correlation between the sex-biased bacteria and microbial metabolites was investigated. Taken together, these findings enhance our understanding of the influences of sex on the microbial community in the different intestinal compartments of Jiangshan black pigs.

The alpha-diversity indices are mainly used to evaluate the variation in microbial diversity. In the present study, we found no differences in alpha-diversity indices (e.g., Chao1, Shannon) between the female and male groups in either the ileum or colon, suggesting that sex exerted minimal influences on the diversity of the microbiota in the intestinal tract. These results differed from those of previous studies on humans and mice, which reported lower gut microbial diversity in males [11,47]. Similarly, in commercial purebred Duroc pigs, entire boars also had a lower fecal microbiota diversity than gilts [14]. These discrepancies may reflect differences in age and the relatively small sample size used in our study [48], which also may have been influenced by diet composition and genetic background [49].

In this study, PCoA revealed the variation in microbial composition between groups, consistent with prior observations in pigs [14]. Meanwhile, many sex-biased bacteria in the gut of Jiangshan black pig were identified in this study. Specifically, in the ileum, the abundances of the genera *Romboutsia* and *Pseudomonas* were higher in the male and female Jiangshan black pigs, respectively. In the colon, the male Jiangshan black pigs showed higher abundances od Unclassified *Lachnospiraceae*, *Eubacterium coprostano-ligenes group*, *Christensenellaceae R-7 group*, *Clostridium_sensu_stricto 1*, and *Ruminococcus* and lower abundances of *Streptococcus*, *Phascolarctobacterium*, *Prevotella* spp., and *Butyricicoccaceae UCG-009*. These results were also supported by a previous study on pigs [50]. In mice, *Lactobacillus*, *Roseburia*, *Eubacterium*, and *Coprococcus* were reported to be the representative sex-biased bacteria [11]; *Eubacterium*, *Blautia* and *Treponema* were identified as sex-biased bacteria in humans [51]. The sex-biased bacteria might be attributed to the differences in sex hormones, such as testosterone and estradiol [7]. It was reported that estrogen inhibited *Escherichia coli* growth in the intestine of rats [52], while this variation was not observed in the present study. Some previous studies showed that, compared with boars, the composition of gut microbiota in castrated male pigs was comparable to that of female pigs [14,50]. Moreover, the circulating testosterone concentration in female mice increased after removing the microbiota, which decreased the concentration in male mice, indicating a bidirectional interaction between male sex hormone levels and microbiota [12].

Functional shifts often accompany compositional changes. The metagenomic functional prediction revealed differences in the microbiota’s gene functions in Jiangshan black pigs between the sexes based on the KEGG database. For example, the metabolic pathways of the gut microbiota enriched in female Jiangshan black pigs were related to amino acid metabolism, such as histidine metabolism; valine, leucine, and isoleucine biosynthesis; phenylalanine, tyrosine, and tryptophan biosynthesis; and tyrosine metabolism. This pattern suggests a stronger microbial capacity for amino acid metabolism in female than in male Jiangshan black pigs. These results might be attributed to increased *Pseudomonas* in the gut of the female pigs. *Pseudomonas* is one member of the Proteobacteria. And Proteobacteria are regarded as the main type of bacteria involved in protein utilization in the gut, which contribute to amino acid metabolism [53]. For the male Jiangshan black pigs, the metabolic pathways enriched in the gut microbiota were related to carbohydrate metabolism (such as pentose and glucuronate interconversions, pentose phosphate pathway, starch and sucrose metabolism) and membrane transport (ABC transporters). These results indicated that the microbiota in the gut of male Jiangshan black pigs had a stronger ability to use carbohydrates in the diet, which might have been associated with the increases in *Eubacterium*, *Ruminococcus*, and *Christensenellaceae R-7 group*. *Eubacterium* and *Ruminococcus* were the primary fiber-degrading bacteria in the gut, promoting the fermentation and utilization of carbohydrates in the diet [54,55]. A previous study showed that *Ruminococcus gnavus* possessed a large number of genes encoding starch- and sucrose-degrading enzymes [56]. Moreover, *Christensenellaceae R-7 group* belongs to the *Christensenellaceae*, which was reported to be a marker of gut health [57]. A study on humans further showed that *Christensenellaceae* was inversely related to host body mass index (BMI), total cholesterol, and low-density lipoprotein, and positively correlated with fiber fermentation and protein catabolism [58]. In summary, although direct performance data were not collected, the observed enrichment in fiber-degrading bacteria in males suggests positive impacts on feed efficiency, especially roughage, which should be confirmed through growth or nutrient utilization studies.

The microbial metabolites are the key indicators reflecting microbiota metabolism activity, such as SCFAs and lactate. In the present study, higher concentrations of propionate, isobutyrate and valerate were found in the gut of the female Jiangshan black pigs, hinting that the bacteria in female pigs exhibited a stronger capacity for SCFA production. These results might have been due to the increases in *Prevotella* spp., *Phascolarctobacterium*, and *Butyricicoccaceae UCG-009*, as *Prevotella* spp. are well-known propionate-producing bacteria in the intestinal tract [59]. Correlation analysis further confirmed these results (as shown in Figure 9). *Phascolarctobacterium* can also utilize succinate to yield propionate [59]. Furthermore, in this study, the gut microbiota in the female Jiangshan black pigs expressed higher copy numbers of functional genes encoding *mmdA*, involved in propionate production [60]. This finding further demonstrated that the microbiota possessed a stronger capacity to produce propionate in the gut of female Jiangshan black pigs. Lactate, another important microbial metabolite, is mainly produced by lactate-producing bacteria. In this study, the female Jiangshan black pigs showed a higher level of lactate in the gut, which was mainly associated with an increase in *Prevotella_9* and *Streptococcus* [61,62]. Additionally, lactate, as a substrate, could be converted to propionate by lactate-utilizing bacteria, like *Megasphaera elsdenii* [63]. *M. elsdenii*, a specific lactate-utilizing bacteria, can convert lactate to propionate via the acrylate metabolic pathway [63]. Meanwhile, the functional gene *LcdA* expressed by lactate-utilizing bacteria plays a vital role in the process of converting lactate to propionate [64]. Interestingly, we found higher copy numbers of *M. elsdenii* and *LcdA* in the gut of female Jiangshan black pigs, which suggested that the gut microbiota promoted the lactate-to-propionate conversion. NH_3_-N, an amino acid metabolism product, is mainly produced by the microbiota through the deamination process. In this study, female Jiangshan black pigs had a higher concentration of NH_3_-N than male pigs, which might have been related to the increase in *Streptococcus* in the gut. Previous studies have shown that *Streptococcus* is the major amino-acid-utilizing bacteria in the gut [53]. In addition, a significant positive correlation between *Prevotella* spp. and NH_3_-N concentration was observed in this study, suggesting that *Prevotella* spp. played an important role in the process of metabolizing amino acids to produce NH_3_-N. Taken together, sex elicited differential effects on the ileal and colonic microbial community and metabolism in Jiangshan black pigs.

## 5. Conclusions

This study systematically investigated the sex-related differences in the gut microbiota composition and metabolism of Jiangshan black pigs. The results revealed that there were distinct sex-associated differences in the microbial structure and composition in the ileum and colon. In microbial metabolism, female Jiangshan black pigs displayed a greater ability to enhance amino acid metabolism and SCFA generation; male Jiangshan black pigs showed stronger carbohydrate metabolism and transport capacities. These findings provide important guidance for the precision feeding of Jiangshan black pigs, suggesting that low-protein diets may be more suitable for sows, while high-fiber diets could benefit boars. Collectively, the present study highlights the sex discrepancies in the microbial community and metabolism in the small and large intestines of Jiangshan black pigs, providing new insights for precisely modulating the gut microbiota community and metabolism according to sex.

## Figures and Tables

**Table 1:** Composition of diet and nutrient levels.

Ingredient	Content (%)	Nutrient Level	Content (%)
Corn	64.00	Digestive energy (MJ/kg)	13.55
Soybean meal	23.00	Crude protein	16.80
Wheat bran	9.35	Neutral detergent fiber	11.98
Soybean oil	0.60	Acid detergent fiber	4.16
L-lysine	0.18		
L-threonine	0.01		
CaHPO4	0.69		
Rock powder	0.87		
Salt	0.30		
Premix ^1^	1.00		

^1^ Supplied the following per kg of diet: 40 IU, vitamin E; 4000 IU, vitamin D_3_; 10,800 IU, vitamin A; 4 mg, vitamin K_3_; 6 mg, vitamin B_1_; 6 mg, vitamin B_6_; 12 mg, vitamin B_2_; 0.05 mg, vitamin B_12_; 0.2 mg, biotin; 2 mg, folic acid; 50 mg, ascorbic acid; 25 mg D-pantothenic acid; 150 mg, Cu; 100 mg, Fe; 100 mg, Zn; 40 mg, Mn; 0.5 mg, I; 0.3 mg, Se.

**Table 2:** The sequence of primers in this study.

Item	The Sequence of Primers (5′-3′)	Reference
Total bacteria	F:CGGTGAATACGTTCYCGG R:GGWTACCTTGTTACGACT	[33]
Firmicutes	F:GGAGYATGTGGTTTAATTCGAAGCA R:AGCTGACGACAACCATGCAC	[34]
Bacteroidota	F:GGARCATGTGGTTTAATTCGATGAT R:AGCTGACGACAACCATGCAG	[35]
*Lactobacillus*	F:AGCAGTAGGGAATCTTCCA R:ATTCCACCGCTACACATG	[36]
*Bifidobacterium*	F:TCGCGTCYGGTGTGAAAG R:GGTGTTCTTCCCGATATCTACA	[37]
*Streptococcus*	F:ACCAGAAAGGGACGGCTAACTAC R:ATCGTTTACGGCGTGGACTAC	This study
*Coprococcus eutactus*	F:TTCCAGTAGCCAGCAGTMAGAT R:CAATCCGAACTGAGACAGCC	[38]
*Faecalibacterium prausnitzii*	F:GGAGGAAGAAGGTCTTCGG R:AATTCCGCCTACCTCTGCACT	[39]
*Megasphaera elsdenii*	F:CGAACGAGAAGAGATGAGAAGC R:TCCTTCAGCGAAAGCTCCGA	[40]
*Escherichia coli*	F:CATGCCGCGTGTATGAAGAA R:CGGGTAACGTCAATGAGCAAA	This study
*mmdA (Bacteriodes)*	F:ATGTTCCTCACCGGACC R:CGCCGATCACTTCGTACA	[41]
*mmdA (Prevotella)*	F:GGTACAGGTCAGGAGTAC R:CAGATRCGGAAACGDGTGT	[41]
*BCoAT*	F:GCIGAICATTTCACITGGAAYWSITGGCAYATG R:CCTGCCTTTGCAATRTCIACRAANGC	[41]
*LcdA*	F:CTGGTGTGCTGGWSIGCIWSIGTIGCNCC R:CAGATAGGTCCAIAYIGCDATNCCYTCCCA	[41]

## Data Availability

The original contributions presented in this study are included in the article. Further inquiries can be directed to the corresponding authors.

## Abbreviations

The following abbreviations are used in this study:

SCFA	Short chain fatty acid
MCP	Microbial protein
ASV	Amplicon sequence variants
LDA	linear discriminant analysis
FDR	False discovery rate
KEGG	Kyoto encyclopedia of genes and genomes
PCoA	Principal coordinate analysis
PLS-DA	Partial least squares discriminant analysis
PCR	Polymerase chain reaction
mmdA	Methylmalonyl-CoA decarboxylase
BCoAT	Butyryl-CoA:acetate CoA-transferase
LcdA	Lactoyl-CoA dehydratase
